# Non-fatal overdoses and related risk factors among people who inject drugs in St. Petersburg, Russia and Kohtla-Järve, Estonia

**DOI:** 10.1186/s12889-015-2604-6

**Published:** 2015-12-18

**Authors:** Anneli Uusküla, Mait Raag, Sigrid Vorobjov, Kristi Rüütel, Alexandra Lyubimova, Olga S. Levina, Robert Heimer

**Affiliations:** Department of Public Health, University of Tartu, Ravila st 19, 50409 Tartu, Estonia; Infectious Diseases and Drug Monitoring Department, National Institute for Health Development, Hiiu 42, 11619 Tallinn, Estonia; NGO Stellit, St Petersburg, Russian Federation; Department of Epidemiology of Microbial Diseases and the Center for Interdisciplinary Research on AIDS, Yale University School of Public Health, 60 College St, New Haven, CT 06510 USA

**Keywords:** People who inject drugs, Overdose, Eastern Europe, Fentanyl, Opioids

## Abstract

**Background:**

This study seeks to identify the prevalence of, and risk factors associated with, non-fatal overdose among people currently injecting drugs (PWID) in St. Petersburg (Russia) and in Kohtla-Järve (Estonia).

**Methods:**

Five hundred eighty-eight study participants in Kohtla-Järve (in 2012) and 811 in St. Petersburg (in 2012–2013) were recruited using respondent driven sampling for interviewing and HIV testing.

**Results:**

Three-quarters (76 %) of the current PWID were male. Participants from St. Petersburg were older (mean age 32.1 vs. 29.6 years, *p* < 0.0001) and reported a longer average duration of injecting drugs (mean duration: 13.3 vs. 10.9 years, *p* < 0.0001). Main drugs injected were opioids (fentanyl in Kohtla-Järve, heroin in St Petersburg). HIV prevalence was 63 % (95 % CI 59–67 %) in Kohtla-Järve and 56 % (95 % CI 52–59 %) in St. Petersburg. Two thirds of the PWID in Kohtla-Järve and St. Petersburg reported ever having experienced a drug overdose involving loss of consciousness or stopping breathing. In Kohtla-Järve, 28 % (95 % CI 24–31 %) of participants and, in St Petersburg, 16 % (95 % CI 14–19 %) of participants reported an overdose within the previous 12 months. Characteristics of injection drug use practice (longer duration of injection drug use, main drug injected), correlates of high-risk injection behaviour (higher injecting frequency, sharing), and problem alcohol use were associated with the risk of overdose within the previous 12 months. The significant factors effects did not differ between the sites.

**Conclusions:**

PWID are at high risk for overdose. Effective overdose prevention efforts at the public health scale are therefore warranted.

## Background

A recent systematic review documented that mortality is much higher in those who inject drugs than in the general population [1]. Drug overdose is the leading cause of death for people who inject drugs (PWID) [2]. Fatal overdose, however, makes up only a small proportion of overdose events, estimated at between 2 % and 4 % [3]. Non-fatal overdose carries significant morbidity [4] and recent studies have identified previous non-fatal overdose experiences as a significant predictor of subsequent non-fatal overdose [5, 6]. Of importance, research has also documented significant overlap between the correlates of fatal [7–11] and non-fatal overdoses [12]. An estimated 3.5 million people (1.5 % of the population) inject drugs in Eastern Europe [13], a consequence of injection drug use epidemics that started in the early 1990s and that fuelled explosive HIV epidemics beginning from the late 1990s and early 2000s. In Russia, overdose is a major cause of premature and preventable death among drug users [14, 15]. One large, multi-city study of PWID conducted in Russia reported that among participating injection drug users, 59 % had experienced an overdose, 81 % reported witnessing an overdose, and 15 % stated that a witnessed overdose had been fatal [16]. For 2012, the average mortality rate due to opioid overdoses in Europe was estimated at 17 deaths per million population aged 15–64, with the highest rate reported from Estonia (19.1 deaths per 100 000) [17]. In Estonia, overdose deaths are mostly related to the use of fentanyls (fentanyl, 3-methyl-fentanyl), which are highly potent synthetic opioids. Overdose mortality statistics from Russia are less accurate, but by some estimates there may be as many as 100,000 overdose deaths each year (equal to 65 deaths per 100,000) [18]. According to information from the Russian Federal Drug Control Service in 2013, 28.7 people out of 100,000 in urban populations died from overdose and drug related diseases [18]. Most overdoses resulted from heroin or a combination of heroin and alcohol, although the recent appearance of methadone (a potent synthetic opioid) as an illegal injected drug has resulted in a tenfold increase in the number of overdoses (both fatal and non-fatal) reported in St. Petersburg between 2010 and 2012 [19, 20].

Less attention has been paid to non-fatal overdoses. Some data on factors contributing to the overdose risk are available in Russia [15, 16, 21] but they are scant for Estonia. In a small sample of 60 PWID from St. Petersburg, Grau et al. (2009) [15] found that 27 (45 %) reported experiencing a non-fatal overdose in the year prior to being interviewed. Walley et al. (2014) [21] found that in clinical populations studied in St. Petersburg, 16 % of HIV infected individual who both injected drugs and were heavy drinkers reported having had a non-fatal overdose in the past 3 months. They also found that higher injection frequency together with being on antiretroviral therapy (ART) were associated with experiencing an overdose.

The timing and trajectory of HIV epidemics in Estonia and Russia have been similar, driven by transmission among people who inject drugs, most commonly opioids. Despite the surface similarities in the nature of their HIV epidemics, structural differences between Russia and Estonia may shape the risk profiles for drug overdose. This includes significant differences in the type of opioids used by PWID in Estonia, where fentanyl is most commonly injected, and St. Petersburg, where heroin injection is most common. In addition, Estonia but not St. Petersburg provides low-threshold HIV prevention and care for people who inject drugs, most notably government-established and funded opioid substitution and harm reduction programs. Specifically, the Estonian system of care has incorporated evidence-based public health practices, including syringe exchange programs, for approximately 10 years, featuring extensive collaboration between the government and non-governmental organizations [22]. It has been estimated that approximately 120 syringes per PWID per year have been distributed since 2008 in Estonia [23]. Antiretroviral treatment (ART) is available free of charge to all patients in need, including those without health insurance. Studies conducted in Estonia have documented that in recent years over 40 % of PWID who have HIV are receiving ART [23].

In contrast, opioid substitution is illegal in Russia, harm reduction is supported neither by the Russian federal government nor most regional governments, and Russian police sometimes interfere with existing harm reduction services [24]. Syringe exchange programs, where they exist, are generally run by non-governmental organizations operating without much political or financial support [25]. Despite the fact that Russia doubled funding for antiretroviral therapy in 2007, in the years that followed, as few as 1 % of people who inject drugs who needed such treatment have received it [26].

The current study sought to identify risk factors associated with non-fatal overdose among current injection drug users in St. Petersburg (Russia) and in Kohtla-Järve (Estonia) who were not recruited from HIV or drug treatment clinics. Both cities are administrative centres for the respective (and adjoining) areas of Estonia (North East County) and Russia (Leningradskaya Oblast). The two sites were selected to obtain additional information on factors related to overdose risk by contrasting findings from different circumstances of drug use and harm reduction approaches in those two sites.

## Methods

### Study sample

At both sites, we conducted cross sectional studies among current PWID defined as persons reporting injecting drugs in the past 4 weeks. Recruitment for an interviewer-administered risk behaviour survey and HIV testing was carried out through respondent-driven sampling (RDS) as described in prior studies [27, 28]. Study participation was anonymous. Participants had to be 18 years or older, speak Russian or (in Estonia) Estonian, and be able to provide informed consent. Surveys were administered by a team of trained fieldworkers, in confidence, in Kohtla-Järve in rooms of the needle and syringe program (NSP) and in St. Petersburg, at 7 independent recruitment locations across regions of the city served by outreach vans. Recruitment began with the non-random selection of a small number of ‘seeds’ representing diverse PWID types (by gender, main type of drug used, and HIV status). A dual incentive approach (primary incentive for participation in the study and a secondary incentive for each eligible person recruited to the study) was used. To assure eligibility, participants were asked to show evidence of injection stigmata. If this was not possible (for example if injections were made in the groin or if the respondent was not a regular injector so that fresh stigmata were not visible) potential participants were asked questions to ascertain their familiarity with injecting drug use practices before the start of the interview. All interviewers were familiar with the process of injecting and were able to discern appropriate responses.

The study questionnaire for both sites was based on the WHO Drug Injecting Study Phase II survey (version 2b(rev.2)) [29] originally developed to collect risk behaviour data from PWID, and therefore contained similar questions on key socio-demographic and behavioural characteristics of drug injection (duration of injecting, injection frequency, main drug injected, multi-drug use, sharing within the last 4 weeks), problem alcohol use (CAGE score) [30], assessment of mental health status (Mental health inventory 5, (MHI-5)) [31, 32], and use of harm reduction and prevention and drug treatment services, history of incarceration, and HIV testing and care. The instrument was modified to obtain information on the illicit drugs known to be available in St. Petersburg (Russia) and in Kohtla-Järve (Estonia), on experienced and witnessed drug overdoses, and on internalized and experienced stigma.

HIV counselling was provided, and a specimen was collected for HIV testing. In Kohtla-Järve, venous blood was tested with commercially available kits for HIV antibodies (ADVIA Centaur HIV Antigen/Antibody Combo Assay, Siemens Healthcare Diagnostics), and for those testing HIV positive on the screening test, the diagnosis was confirmed using INNO LIA HIV I/II Score Western blot (Fujirebio Europe) and results were reported back to participants). In St. Petersburg, rapid oral HIV testing was used (OraQuick ADVANCE® Rapid HIV-1/2 Antibody Test, OraSure Technologies Inc.) and those testing positive were referred to the City AIDS Center for confirmatory testing.

In Estonia, data collection was conducted from June to August 2012 and in St Petersburg from November 2012 to June 2013.

Ethical approval was obtained from the Ethics Review Board of the University of Tartu (Estonia), the Institutional Review Board at NGO Stellit in St. Petersburg (Russian Federation), and the Human Investigation Committee at Yale University (USA).

### Data analysis

Descriptive statistics were used to summarize the data by site. The Pearson chi-squared test (for categorical variables) and *t*-test with unequal variances (for continuous variables with normal distribution) or Wilcoxon rank-sum test (for continuous variables with non-normal distribution) were used to explore differences between the sites.

The proportion of participants self-reporting non-fatal overdose within the last 12 months (defined in the questionnaire as having “lost consciousness or stopped breathing as a result of taking drugs”) was calculated. This overdose definition probably restricts the accounts to opioid-related overdose [33]. This focus was made to acknowledge the fact that the overwhelming majority (>90 %) of deaths related to injection drug use in the regions studied are related to the opioids use [16, 34, 35]

Factors associated with report of non-fatal overdose within the last 12 months were examined separately for both of the sites using univariable and multivariable logistic regression, from which odds ratios (OR) and adjusted OR (AOR), with corresponding 95 % confidence intervals (CIs), were calculated. In addition to age and gender, all factors associated with the outcome variable in the univariate analysis at *p* < 0.05 at either site were included in multivariable models. So, the same factors were included in both multiple regression models, to promote comparison between sites. Due to different drug profiles, main drug for injecting was dichotomised for both sites (opioids vs. other) to facilitate comparison of multivariable analysis between the sites. We further tested whether the significant factors effects were significantly different for the sites by providing ratios of respective ORs with corresponding 95 % CIs. We determined statistical significance using the penalized maximum likelihood ratio test statistic [36].

## Results

### Sample characteristics by city

Table 1 presents the characteristics of the 588 study participants recruited in Kohtla-Järve and 811 in St. Petersburg by city. Across the two sites, three quarters of the current PWID were male. Participants from St. Petersburg tended to be older (*p* < 0.0001).

**Table 1:** Socio-demographic, injection drug use, HIV prevalence and care and environmental characteristics of current injection drug users participating and the prevalence of self-reported non lethal overdose within the last 12 months from cross sectional studies in Kohtla-Järve, Estonia (in 2012) and St Petersburg, Russia (in 2012 – 2013)

	Kohtla-Järve (Estonia), *N* = 588			St Petersburg (Russia), *N* = 811		
Characteristics	*n*	%	Overdose last 12 months (%)	*n*	%	Overdose last 12 months (%)
*Socio-demographic indicators*						
Age			*p* = 0.134			*p* = 0.337
29 or less	295	50 %	25 %	245	30 %	18 %
30 or more	293	50 %	30 %	566	70 %	15 %
Gender			*p* = 0.833			*p* = 0.423
Male	435	74 %	27 %	631	78 %	16 %
Female	152	26 %	28 %	180	22 %	18 %
Main source of income (last 6 months)			*p* = 0.239			*p* = 0.021
Work (full/part time)	192	33 %	23 %	590	73 %	14 %
Social benefits	263	45 %	30 %	22	3 %	14 %
Other	127	22 %	31 %	199	25 %	23 %
*Drug use characteristics*						
Duration of injecting			*p* < 0.001			*p* = 0.008
< = 9 years	230	39 %	16 %	170	21 %	9 %
= > 10 years	356	61 %	35 %	640	79 %	18 %
Frequency of injecting (last 4 weeks)			*p* < 0.001			*p* < 0.001
< Daily	447	76 %	24 %	524	65 %	11 %
Daily +	139	24 %	40 %	287	35 %	26 %
Main drug injected (last 4 weeks)			*p* < 0.001			*p* = 0.116
Amphetamine	195	35 %	14 %	27	3 %	4 %
Fentanyl	350	62 %	34 %	4	0 %	25 %
Heroin	1	0 %		563	69 %	18 %
Methadone	0	0 %		217	27 %	14 %
Injecting multiple drugs (last 4 weeks)			*p* = 0.124			*p* = 0.003
No	378	64 %	25 %	495	61 %	8 %
Yes	210	36 %	31 %	316	39 %	21 %
Non-injecting drug use (last 4 weeks)			*p* = 0.575			*p* = 0.037
No	324	57 %	27 %	755	93 %	15 %
Yes	246	43 %	29 %	56	7 %	27 %
Sharing (last 4 weeks)			*p* < 0.001			*p* < 0.001
* No*	492	85 %	24 %	277	34 %	7 %
* Yes*	88	15 %	49 %	534	66 %	21 %
*Problem alcohol use*			*p* = 0.009			*p* < 0.001
* No*	247	42 %	22 %	275	34 %	8 %
* Yes*	341	58 %	32 %	536	66 %	21 %
*Mental health (MHI 5)*			*p* = 0.003			*p* < 0.001
* > = 52*	362	62 %	23 %	536	66 %	13 %
* <52*	221	38 %	35 %	275	34 %	23 %
*Structural (environmental) characteristics*						
Homelessness (current)			*p* = 0.619			*p* = 0.454
No	583	99 %	27 %	798	98 %	16 %
Yes	5	1 %	40 %	13	2 %	14 %
Ever been in prison			*p* < 0.001			*p* = 0.368
No	266	45 %	19 %	537	66 %	17 %
Yes	322	55 %	35 %	274	34 %	15 %
Main source of syringes (last 4 weeks)			*p* = 0.0018			*p* = 0.0121
Needle and syringe program (NSP)	445	77 %	30 %	119	15 %	11 %
Pharmacy	76	13 %	16 %	615	76 %	19 %
Other	26	5 %	35 %	30	4 %	10 %
None	29	5 %	7 %	46	6 %	4 %
Currently receiving drug treatment			*p* > 0.9			*p* = 0.133
No	216	68 %	32 %	563	97 %	18 %
Yes	102	32 %	32 %	19	3 %	32 %
*HIV infection and care*						
HIV infected			*p* = 0.013			*p* = 0.021
No	218	37 %	22 %	359	44 %	13 %
Yes	370	63 %	31 %	452	56 %	19 %
Currently on ART			*p* = 0.342			*p* = 0.051
No	247	67 %	33 %	403	90 %	20 %
Yes	123	33 %	28 %	47	10 %	9 %

The participants in St. Petersburg reported a significantly longer duration of injecting drugs (*p* < 0.0001), and the proportion of those injecting daily or more frequently was higher in St. Petersburg (*p* < 0.0001). For both sites opioids were the main injection drugs, however in Kohtla-Järve the synthetic opioid fentanyl was the most common main drug injected (reported by 62 %, followed by amphetamine, 35 %) whereas in St. Petersburg, heroin was named as the main drug for injecting by 69 % of participants (followed by another opioid, methadone, 27 %). (Of note, fentanyl injected in Kohtla-Järve and methadone injected in St. Petersburg are not diverted prescription medicines; these products appear to be locally synthesized directly for sale in illegal markets). Slightly over one-third of PWIDs at both sites reported recent injections of more than one drug. Concurrent with injection, consumption of drugs by other means was significantly more frequent in Kohtla-Järve than in St Petersburg (*p* < 0.0001). In Estonia, of those reporting non-injection drug use in addition to injecting (*n* = 246), oral use was reported by 73 % for amphetamine, 50 % for methylenedioxy-methamphetamine (“ecstasy”), 24 % for fentanyl, 22 % for cocaine, and 15 % for methamphetamine. Few respondents reported using benzodiazepines (*n* = 10). In St Petersburg, the main non-injected substances used were heroin and amphetamine (reported by 45 % and 79 %, respectively, of the 56 people using drugs other than by injection).

Two-thirds of the respondents reported problem alcohol use (according to the CAGE score) and one third of the sample reported clinically significant psychological distress/mental health problems (MHI 5 scores < 52) [32].

The proportion of participants who had been in prison was higher in Kohtla-Järve (*p* < 0.0001) as was the proportion of those receiving drug treatment at the time of the study (*p* < 0.0001). Of the participants in drug treatment during the study participation in Estonia, the majority (92 %) were receiving methadone substitution treatment.

Based on testing, HIV prevalence was 63 % (95 % CI 59–67 %) in Kohtla-Järve and 56 % (95 % CI 52–59 %) in St. Petersburg. Of those who were HIV-positive, 33 % were on ART in Kohtla-Järve compared with 10 % in St. Petersburg (*p* < 0.0001).

Two-thirds of the PWID in Kohtla-Järve (*n* = 377) and St. Petersburg (*n* = 527) (64 % and 65 %, respectively) reported ever having had a drug overdose involving loss of consciousness or stopping breathing. In Kohtla-Järve, 162 (28 %, 95 % CI 24–31 %) participants and in St Petersburg, 132 (16 %, 95 % CI 14–19 %) of participants reported having a drug overdose within the previous 12 months (*p* < 0.0001).

### Risk factors associated with non-lethal overdose within the last 12 months

#### Univariate analysis

The analysis for both cities are presented in Table 1.

In Kohtla-Järve, increased odds of overdose in the last 12 months were associated with several factors in the univariate analyses: longer duration of injecting (over 10 years: OR 2.8 95 % CI 1.3–4.3), injecting daily (vs. less than daily: OR 2.2, 95 % CI 1.4–3.3), injecting fentanyl (vs. amphetamine: OR 3.2 95 % CI 2.0–5.3), sharing syringes (OR 3.03, 95 % CI 1.84–4.95), ever being in prison (OR 2.3 95 % CI 1.5–3.5), and being HIV infected (OR 1.6 95 % CI 1.1–2.5). Further, problem alcohol use (OR 1.66, 95 % CI 1.12–2.47) and current mental health problems (OR 1.77, 95 % CI 1.20–2.60) were associated with increased odds for overdose. Reporting pharmacy as the main source of syringes in last 4 weeks was associated with decreased odds for overdose (vs NSP: OR 0.44 95 % CI 0.21–0.85).

In St. Petersburg, increased odds of overdose were associated with the duration of injecting (over 10 years: OR 2.1 95 % CI 1.2–4.0), injecting daily (OR 2.9, 95 % CI 2.0–4.3), injecting multiple drugs (OR 1.8 95 % CI 1.2–2.6), reporting non-injecting drug use (OR 2.0 95 % CI 1.0–3.8), sharing syringes (OR 3.41, 95 % CI 2.04–5.94), reporting pharmacy as the main source of syringes (vs NSP OR 1.9 95 % CI 1.0–3.7), being HIV infected (OR 1.6, 95 % CI 1.1–2.4). As in Estonia, both problem alcohol use (OR 3.15, 95 % CI 1.91–5.44) and prevalent mental health problems (OR 2.09, 95 % CI 1.40–3.10) were associated with increased odds for overdose.

### Multivariable analysis

Table 2 presents the factors associated with the risk of overdose within the 12 months prior to interview in multivariable modelling. The significant factors effects did not differ for the two sites. In Kohtla-Järve these were longer duration of injection drug use (over 10 years: AOR 2.77, 95 % CI 1.55–5.08), main drug of injection (fentanyl vs. amphetamine: AOR 1.70, 95 % CI 1.06–2.72), and sharing (AOR 2.65, 95 % CI 1.6–4.40) in addition to problem alcohol use (AOR 1.92, 95 % CI 1.25–2.96). In St Petersburg, frequent injecting (AOR 1.75, 95 % CI 1.14–2.69), sharing (AOR 3.09, 95 % CI 1.84–5.40), and problem alcohol use (AOR 2.23, 95 % CI 1.35–3.81) were associated with increased odds for overdose. The point estimate for the overdose association with the use of heroin or methadone as the main drug of injection (vs amphetamine) – AOR 1.6 (95 % CI 0.36–15.39) – was quite similar to the findings from Kohtla-Järve. However, the wide confidence interval, related to the low number of observations in the reference category of ‘amphetamine’ users, precluded finding a confidence interval for the AOR that did not include 1.0.

**Table 2:** Results of multivariable analysis: factors associated with self-reported non fatal overdose within the last 12 months among current injection drug users in Kohtla-Järve, Estonia (in 2012) and St Petersburg, Russia (in 2012–2013)

	Kohtla-Järve (Estonia)	St Petersburg (Russia)	AOR_KJ_/AOR_SP_
	AOR* (95 % CI)	AOR* (95 % CI)	
Duration of injecting (10+ years)	2.77 (1.55–5.08)***	1.59 (0.87–3.03)	1.75 (0.72–4.23)
Injecting frequency (daily)	1.44 (0.91–2.27)	1.75 (1.14–2.69)**	0.82 (0.44–1.54)
Main drug injected (opioid)^1^	1.70 (1.06–2.72)**	1.61 (0.36–15.39)	1.05 (0.10–10.60)
Sharing	2.65 (1.60–4.40)***	3.09 (1.84–5.40)***	0.86 (0.40–1.83)
Problem alcohol use	1.92 (1.25–2.96)***	2.23 (1.35–3.81) ***	0.86 (0.43–1.72)

*OR adjusted for age, sex, main source of income last 6 months, injecting multiple drugs last 4 weeks, non-injecting drug use last 4 weeks, ever been in prison, ever received drug treatment, main source of syringes last 4 weeks, ever tested for HIV, HIV infected

**p < 0.05, ***p < 0.01

^1^Using fentanyl or heroin in Kohtla-Järve and fentanyl, heroin or methadone in St. Petersburg defined as ‘opioids as main drug used’ (vs amphetamine use as a reference group at both sites)

## Discussion

This project investigated a subject that has been insufficiently studied in the countries of the former Soviet Union: the occurrence of non-fatal overdose and factors associated with this experience within the last 12 months among current PWID. Two-thirds of current injectors reported ever having experienced a non-fatal overdose with a significantly higher proportion of PWID in Kohtla-Järve, Estonia (28 %) than in St. Petersburg, Russia (16 %) reporting a non-fatal overdose occurring within the last year.

Although results from studies of non-fatal overdose across countries are often not easily comparable, as they may recruit drug users from different settings (population based or clinical service based), target populations (heroin, fentanyl, or pharmaceutical opioid users, problem drug users, injectors non-injectors), and use different recall periods, we have used the same method for sample recruitment and the same instrument to collect interview data at contiguous sites separated by a national border and local differences in the types of drugs injected. In attempting to compare our results to previous studies from a range of countries, we found reports in the literature for lifetime overdose histories ranging between one-third and two-thirds of heroin users [36]. Our finding that two thirds of current injectors reported having experienced a non-fatal overdose is clearly at the higher end of the spectrum. On the other hand, the high proportion of PWID reporting non-fatal overdose is in agreement with the high overdose related mortality reported from both Estonia and the Russian Federation.

We also assessed the factors associated with recent non-fatal overdoses (within the last 12 months). Our finding of a higher risk of overdose among long-term injectors is in agreement with findings from other studies [4, 12, 37]. The structural factors, even though different at the two sites, were not associated with overdose risk in our analysis. Characteristics of injection drug use practice (duration, injection frequency, main drug injected), correlates of high-risk injection behaviour (sharing) and problem alcohol use were associated with higher odds for overdose, and significant factors effects did not differ for the sites.

Higher overdose rates among those injecting more frequently or for more years [21, 38, 39], as well as among those PWID exhibiting high-risk injection behaviour (sharing syringes), have been described before [40].

A significantly higher proportion of participants reported overdose experiences in the past 12 months in Estonia. There are several potential interpretations of this finding. Fluctuations in illicit drug formulations or purity can have a significant impact on overdose – especially if periods of low purity alternate with episodes of high purity. While there were some significant differences in the socio-demographic and service-utilization characteristics between the sites, these seem unlikely explanations for the observed differences in the 12-month overdose risk. Of the contributors to the overdose risk described in our analysis the (main injected) drugs of choice were significantly different between sites.

In our study, people who reported injecting fentanyl as their main drug were most likely to report experiencing overdose. In the European Union, Estonia has the longest documented epidemic of fentanyl injection among drug-using populations and formulations of fentanyl remain the main injected drugs in Estonia [41, 42]. However, fentanyl use and the related high levels of mortality linked with fentanyl are not limited to Estonia. In 2010 and 2011, following heroin shortages, Bulgaria and Slovakia identified increases in fentanyl use among opioid injectors [41]. Increases in overdose deaths linked with use of fentanyl have been reported by Germany [41] and in multiple locations in the USA [43, 44]. Heroin dominance was first observed in Estonia in the early 2000s but was replaced by fentanyl by the mid 2000s [45]. Heroin dominance, which has been sustained in Russia for some time, has been challenged within the last few years with the emergence of methadone as an illicitly injected drug. If this tendency continues, and there are further changes in the Russian drug scene, more attention to a potential increase in overdose rates is needed from the professional community. We acknowledge that using information on the main drug injected in the last 4 weeks in the analysis measuring the association of the drug of choice with the overdose reported over the past 12 months has its limitations. Some of the overdoses reported by participants injecting mainly amphetamines in the last 4 weeks may have been among those who had also taken opioids during the last 12 months (and thus have had an opioid overdose).

The substantial role of alcohol needs to be highlighted, acknowledging both the high prevalence of problem alcohol use among current injectors and the relatively strong effect for its association with overdose. The prevalence of problem alcohol use among the male urban general population in Russia measured using CAGE scores is reportedly high (23 %) [46], but it was almost 3 times higher among the current injection drug users participating in our study (at both sites). Higher overdose rates associated with alcohol use among injection drug users have been described in other studies [47, 48] and may represent pharmacological or behavioral interactions between alcohol and injected substances [36].

We acknowledge that our study has limitations. First of all, we are describing factors associated with non-fatal overdose; these might differ from factors that increase the likelihood of lethal overdoses. The cross-sectional study design does not allow us to establish causal relationships nor the direction of the associations described. The results of the study may have been affected by recall and social desirability biases. To diminish these potential biases study participation was anonymous and study surveys were conducted by trained interviewers in an environment familiar to the respondents. Although we used a recruitment methodology (RDS) that has been shown to provide an efficient sampling technique for hard-to-reach groups we cannot claim representativeness of the study samples.

While these limitations are important, we feel it is extremely unlikely that they accounted for the patterns that we observed in the data. Our data were generated using similar study methods (including recruitment and measures) and our findings strongly suggest that, in Kohtla-Järve and in St Petersburg, current PWID are at high risk for overdose, exhibit high injection risk behaviours which, as well as being associated with the risk of overdose, also contribute to transmission of HIV and other blood borne infections.

## Conclusions

Our results highlight the relevance of the role of drug of choice and alcohol abuse for observed overdose patterns. Effective and combined overdose prevention efforts that also address problem alcohol use and co-occurring mental health problems, at the public health scale, are warranted as part of comprehensive harm reduction measures.
